# A Nail in the Brain

**DOI:** 10.7759/cureus.51974

**Published:** 2024-01-09

**Authors:** Tze Huei Kee, Khoo Chung Lee, Adlina Abdul Rahim, Noor Khairul Binti Rasid

**Affiliations:** 1 Ophthalmology, Hospital Sultanah Bahiyah, Alor Setar, MYS

**Keywords:** transorbital, perforating eye injury, nail gun, intracranial foreign body, industrial injury

## Abstract

Transorbital penetrating brain injuries (TOPI) are rare. We report a case of industrial injury that resulted in perforating eye injury and intracranial foreign body by a nail gun. A 30-year-old man accidentally fired a nail gun onto his left eye at his construction workplace while handling the malfunctioned equipment and sustained a perforating injury of the left eye with intracranial foreign body. The misfired nail was lodged in his frontal lobe of the brain. He also suffered laceration wounds of the lateral canthus of the left eye and fractures of the left orbital floor and roof. He underwent emergency bicoronal craniotomy and removal of intracranial foreign body, followed by left eye examination under anaesthesia as well as scleral toilet and suturing. The nail was successfully removed. He recovered well with no neurological deficit and was discharged on postoperative day 5 with a Glasgow Coma Scale score of 15; however, his left eye vision remained no perception of light. Work-related eye injuries can be debilitating and are largely preventable.

## Introduction

Penetrating brain injury is not common, accounting for 0.4% of head injuries [1]. Transorbital penetrating brain injury (TOPI) is even rarer, accounting for 24% of penetrating head trauma in adults and 45% in children [2]. Although rare, TOPI can cause serious neurological and ophthalmic disabilities. We herein report a case of industrial injury that resulted in perforating eye injury and intracranial foreign body by a nail gun.

This case report was presented as a poster at the 11th COSC UM - APOT Ophthalmic Trauma Meeting 2022 on September 17, 2022.

## Case presentation

A 30-year-old man, a foreign construction worker, presented to the hospital with loss of vision and bleeding of the left eye following an accident that occurred at his construction workplace. He was using a pneumatic nail gun without wearing protective goggles. When the nail gun jammed, he checked it through the gun barrel and accidentally fired the nail gun onto his left eye.

Upon arrival to the hospital, his Glasgow Coma Scale (GCS) score was 15, and vital signs were stable. Other than left eye wounds and swelling, primary and secondary examinations did not reveal additional injuries. He had no past significant medical, surgical, or drug history. He complained of headache and left eye pain, and otherwise he was cooperative and fully oriented during the examination. The muscular strength and tension of all four limbs were normal. His blood analyses and other biochemical parameters were within normal limits. He was given antitetanus injection, intravenous broad-spectrum antibiotics, and anticonvulsant medication.

**Figure 1:**
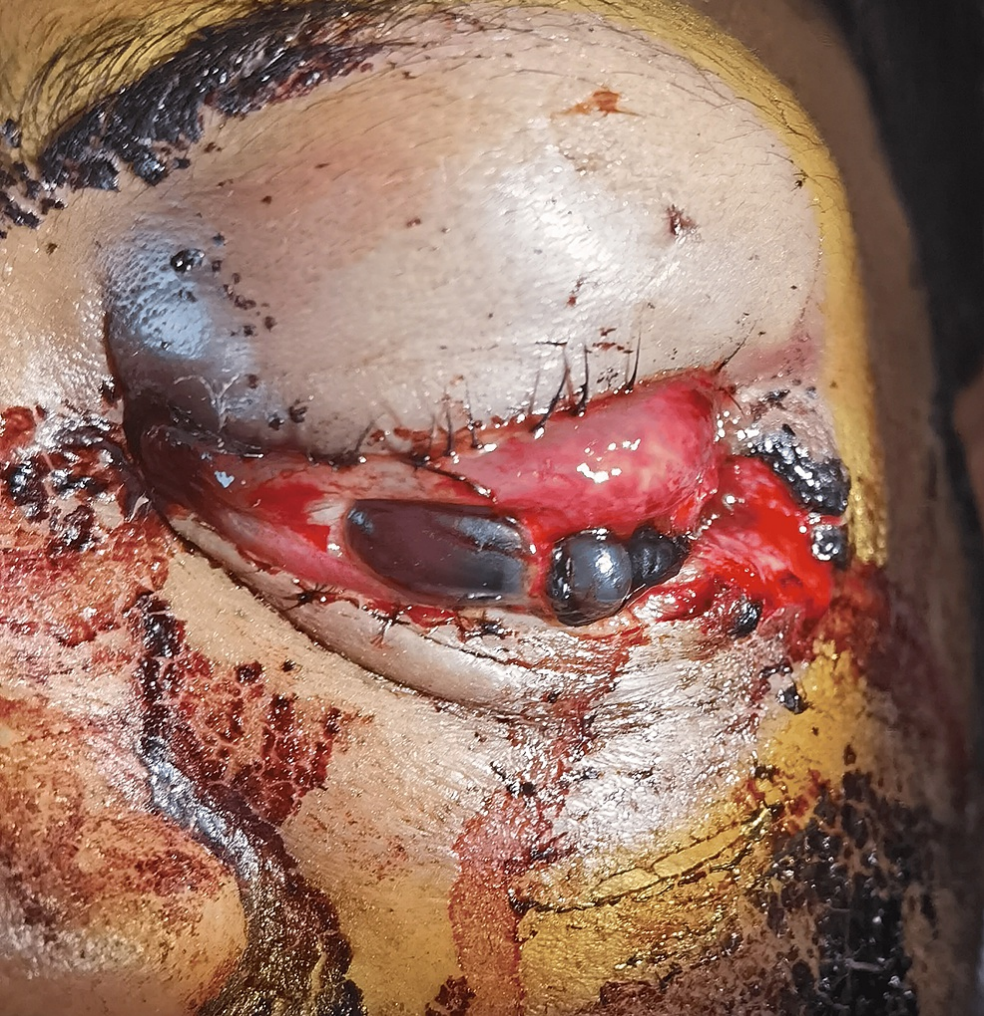
Picture of the patient’s left eye at presentation showing lacerated lateral canthus, prolapse of the uveal tissues, extensive subconjunctival hemorrhage, chemosis, and total hyphema.

Examination of the left eye revealed left eye proptosis with laceration wounds on the lateral canthus. Vision of the left eye was no perception light (NPL). There was left periorbital hematoma, extensive subconjunctival hemorrhage and chemosis, prolapse of uveal tissue from the temporal side of the eyeball, and total hyphema, which obscured visualization of the pupil (Figure 1). Reverse relative afferent pupillary defect (RAPD) of the left eye was positive. Examination of the right eye was unremarkable.

**Figure 2:**
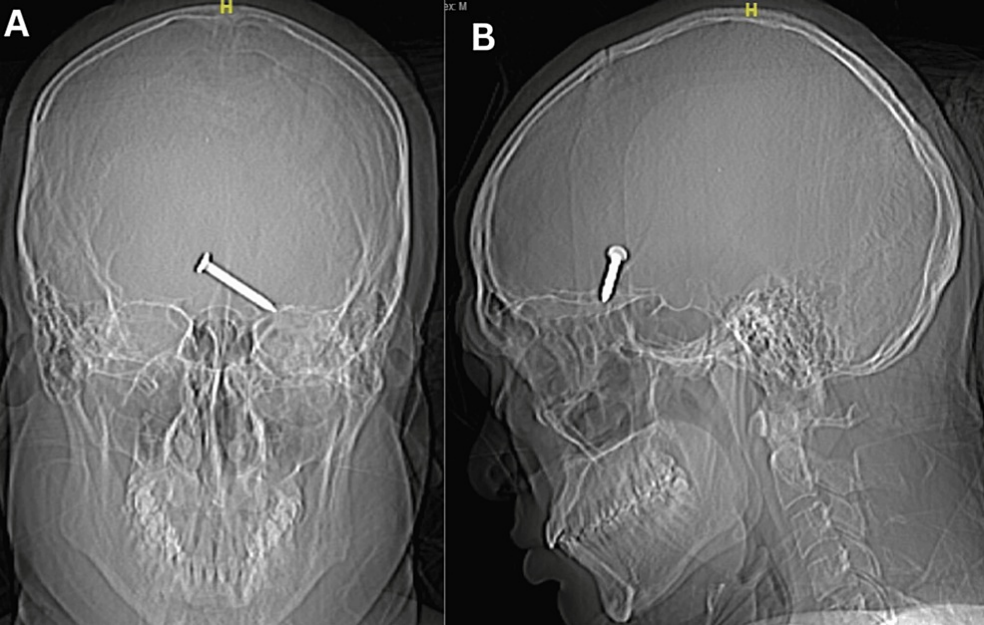
Plain X-ray of the skull, anteroposterior (A) and lateral (B) views, showing a metallic foreign body resembling a nail.

**Figure 3:**
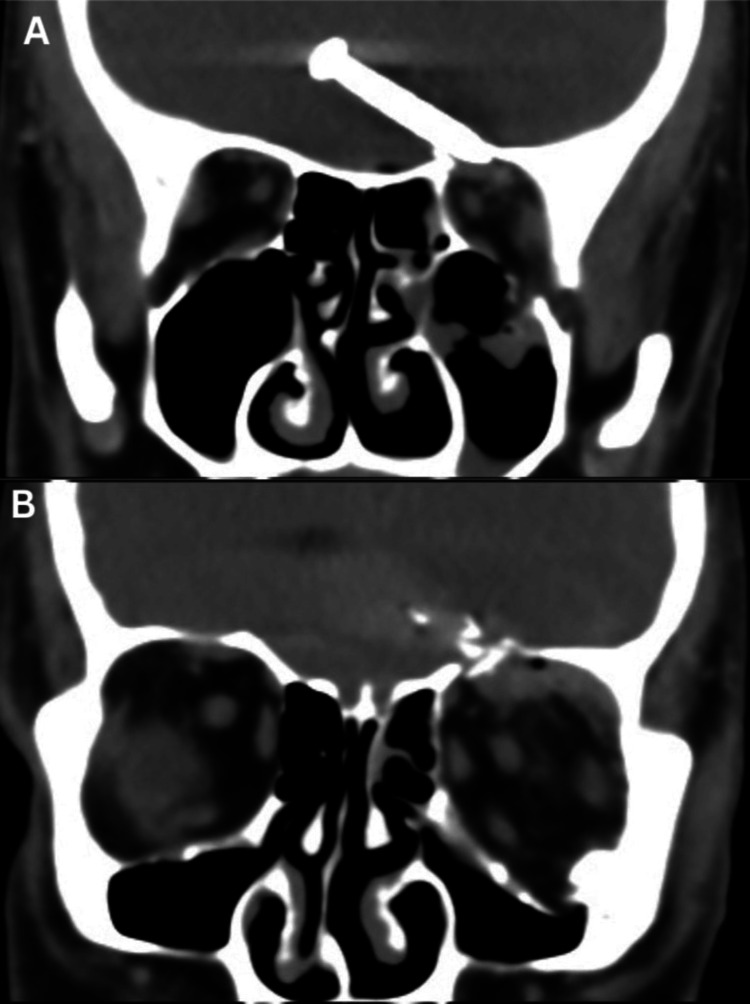
Non-contrasted CT brain (coronal view) showing the nail (A) and fractures of the left orbital roof and floor (B).

**Figure 4:**
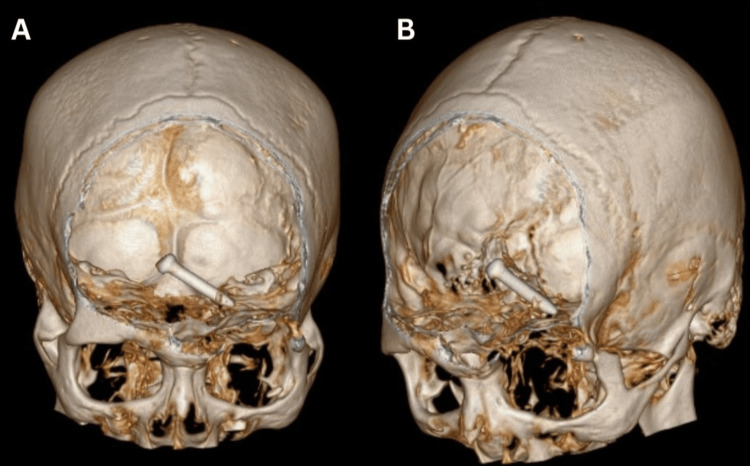
A three-dimensional reconstructed CT of the brain showing the nail penetrated in the left orbital roof and lodged in the frontal lobes.

Plain skull X-ray showed a nail that had penetrated the left orbital roof (Figure 2). The nail, measuring 3.2 cm, was lodged in the frontal lobe of the brain as revealed by plain computed tomography (CT) (Figure 3). There was also the presence of subdural and subarachnoid hemorrhages at the left frontotemporal region extending to the left parietal region. There were fractures of the left orbital roof and floor (Figure 4).

He sustained perforating injury of the left eye with the misfired nail being lodged in the frontal lobe, laceration wounds of the lateral canthus of the left eye, and fractures of the left orbital roof and floor. The patient was co-managed with neurosurgical team and underwent emergency bicoronal craniotomy and removal of intracranial foreign body. Intraoperatively, the nail was found to be penetrated in the parenchyma of bilateral frontal lobes of the brain. The left orbital roof was fragmented with bony pieces that pierced through the dura and brain parenchyma. However, the left internal carotid artery, anterior cerebral arteries, and the olfactory nerves were not injured. The nail was successfully removed as a single piece, and there was no active bleeding after removal of the nail. The surgery was immediately followed by left eye examination under anesthesia, as well as scleral and lid repair. Intraoperatively, there were extensive extrusion of the vitreous and uveal tissue and the crystalline lens via the scleral laceration wound, which measured 10 mm vertically and extended 17 mm posteriorly. The wound was tracked as far as attainable, and the superior and superonasal parts of the globe were explored; no other laceration wound was found. The extruded and non-viable content of the globe was removed, and the scleral wound was closed with 7/0 absorbable sutures. However, severe proptosis due to retro-orbital edema had precluded the apposition of the severely macerated lateral canthus.

Postoperatively, the patient was admitted to the intensive care unit, where intravenous ceftriaxone, metronidazole, and anticonvulsants were administered. There was no cerebrospinal fluid leakage or active bleeding. To prevent exposure keratopathy due to severe proptosis and lagophthalmos, a moisture chamber was created for him. This was prepared using transparent film waterproof dressing covering the left eye with topical chloramphenicol eyedrops and ointment. Daily dressing of the left eye was done. He was extubated on the next day.

He recovered well during his postoperative period with no neurological deficit and was discharged on postoperative day 5 with a GCS score of 15. His left eye proptosis and lagophthalmos improved with fairly clear cornea and hyphema. However, his left eye vision remained NPL. He was discharged with the advice of continuing the moisture chamber. He was seen after one week in the eye clinic. Proptosis and chemosis had much reduced, eyeball was soft, but there was still lagophthalmos. The cornea was fairly clear without signs of exposure keratopathy, and the anterior chamber was formed with hyphema. The left lateral canthal wound was clean, and he was planned for lateral canthal reconstruction. The patient was counselled regarding monocular precautions and was advised to wear protective goggles to prevent any inadvertent trauma to the healthy eye. Unfortunately, the patient was lost to follow-up as he had returned to his native country to continue treatment.

## Discussion

Nail gun is a popular tool in the construction industry as it can significantly increase productivity. The firing speed of a nail gun ranges from 45.7m/s (pneumatic nail gun) to 426.7 m/s (powder-actuated tool) [3]. The ease and speed of nailing enhances productivity at the cost of increased potential for traumatic injury. TOPI has the tendency to occur in young males, resulting in a high risk of blindness and subsequently increasing our economic burden. Three common routes for TOPI to occur are the optic canal, the superior orbital fissure, and the orbital roof, with the thin and fragile orbital roof being the most frequent route, resulting in frontal lobe contusion [4]. TOPIs are mostly caused by missile injuries, gunshot wounds, and shrapnel wounds. Non-missile injuries are often caused by high-velocity sharpened objects, especially metallic materials such as scissors, screwdrivers, knives, or even wooden sticks [5].

Plain skull X-ray in this case confirmed the presence of the nail intracranially. However, plain skull X-ray has a very high failure rate for detecting fractures and certain types of foreign bodies such as wood, plastic, and glass [6]. As such, the gold standard imaging modality for initial radiological assessment in TOPI cases is non-contrasted CT [7]. CT should be performed as soon as possible because it can identify and localize the foreign bodies, assess extension of the lesion and pathway of penetration, and identify bone fragments and hematoma. This provides essential information for planning patient's management and surgical procedures [7]. Three-dimensional CT image of the patient’s skull is also a useful adjunct to surgical planning, as it provides a detailed three-dimensional analysis of the bony pathology images, position, and trajectory of the foreign body.

Surgical repair of the eyelids in this case had been very challenging due to severe chemosis and swelling of periorbital tissues on top of the severely macerated wounds of the eyelids with tissue loss. Although primary closure of the eyelid lacerations was performed in this case, severe proptosis postoperatively had precluded apposition of upper and lower lids, causing total exposure of the cornea with prolapse of conjunctiva. Therefore, to prevent exposure keratopathy, a moisture chamber was applied round the clock with chloramphenicol ointment as a lubricant. Moisture chamber was created using transparent adhesive film dressing. It protects the ocular surface by acting as a barrier against evaporation of tears, thereby increasing the periocular humidity and tear-film lipid-layer thickness [8]. In addition, it can act as a physical barrier to microorganisms and reduce transmission of infection to the eye. The adhesive film dressing was cut to a size sufficient to cover the area from the eyebrow to the cheek vertically and from the nasal bone to the lateral orbital rim horizontally and was replaced daily. Moisture chamber is an effective measure to preserve the corneal surface temporarily while awaiting definitive surgical repair of the eyelids.

There are various measures to prevent exposure keratopathy include regular eye toileting, frequent lubrication (ointment and drops), lid taping, transparent hydrogel dressings, and moisture chambers with swimming goggles or polyethylene covers. A systematic review and meta-analysis by Zhou et al. found that moisture chambers are significantly better than lubrication without using a moisture chamber in preventing exposure keratopathy in intensive care unit patients [9]. A randomized controlled study by Shan and Min found that the moisture chambers using polyethylene film covers were statistically significantly more likely to prevent exposure keratopathy than artificial tears alone for intensive care patients. Moreover, it also showed that the use of polyethylene film covers was also more time-saving compared to artificial tears alone [10].

## Conclusions

In conclusion, TOPI is an emergency that requires immediate multidisciplinary workup and surgical intervention. Work-related eye injuries especially TOPI can be debilitating and potentially life-threatening, which, in turn, causes productivity loss and increases economic burden. However, they are largely preventable by increasing awareness and providing adequate education on workplace safety measures. This case highlights the importance of wearing proper personal protective equipment at work, which includes protective eye wear such as safety goggles with polycarbonate lenses and face shields, head protection such as helmets, respiratory protection such as respirators and masks, and hand and body protection such as gloves, vests, aprons, and boots. Adequate personal protective equipment should be provided by the employers, as well as proper training and demonstration on safe handling of the tools for the workers in order to prevent work-related injuries.
